# Microsurgery for brain aneurysms in an accessory A2 and basilar arteries: a rare case presentation and surgical video

**DOI:** 10.1093/jscr/rjad742

**Published:** 2024-01-16

**Authors:** Jhon E Bocanegra-Becerra, José Luis Acha Sánchez, Luis Contreras Montenegro

**Affiliations:** School of Medicine, Universidad Peruana Cayetano Heredia, Lima, Peru; Vascular Neurosurgery and Skull Base Division, Department of Neurosurgery, Hospital Nacional Dos de Mayo, Lima, Peru; School of Medicine, Universidad Nacional Mayor de San Marcos, Lima, Peru; Vascular Neurosurgery and Skull Base Division, Department of Neurosurgery, Hospital Nacional Dos de Mayo, Lima, Peru; School of Medicine, Universidad Nacional Mayor de San Marcos, Lima, Peru

**Keywords:** accessory A2 artery, basilar artery, brain aneurysm, median artery of the corpus callosum, microsurgery, surgical video

## Abstract

We present the case of a 58-year-old male with a 3-day history of sudden onset headache, loss of consciousness, and uncontrolled vomiting. The patient had 3/5 quadriparesis and a Glasgow coma scale (GCS) score of 8, which merited neurocritical intensive care. Brain imaging suggested the presence of two lesions: (i) a fusiform aneurysm of 12 × 7 mm in an accessory A2 artery of the anterior cerebral artery and (ii) an unruptured saccular aneurysm of 3.3 × 2.8 mm in the distal segment of the basilar artery. He was deemed a candidate for microsurgical management. Postoperatively, he had 4/5 quadriparesis, paresis of the right oculomotor nerve, and a GCS score of 13. A 3-month follow-up showed a significant improvement in neurological function with a score of 2 on the modified Rankin scale. The presented case illustrates the relevance of a nuanced acquaintance to operate in diseased anatomical variants and complex pathologies of narrow corridors.

## Introduction

The anterior cerebral circulation is supplied primordially by the anterior cerebral artery (ACA) and the middle cerebral artery [1]. Arising from the internal carotid artery (ICA), the ACA is a paired structure that connects each other through the anterior communicating artery (ACoA) and continues independently. However, during embryological development, ACA anatomy variations can occur. An accessory A2 segment is believed to result from (i) the persistence of the embryonic median artery of the corpus callosum or (ii) duplication of one of the ACAs [2–4]. This variant has received several names including medial ACA, the median callosal artery, the median artery of the corpus callosum, the superior callosal artery, third A2 artery, triplicate A2 artery, and accessory ACA. Stemming from the ACoA complex, this vessel can arise as a single or paired structure, thus doting the prefixes of unihemispheric or bihemispheric, respectively [1, 5].

The prevalence of an accessory A2 artery varies between 1% and 13% [1, 2, 6–12]. Prominently, this variant has surgical relevance in the context of aneurysms in the ACoA complex, given the potential supply to the corpus callosum, adjacent cortex, septal nuclei, septum pellucidum, and upper portion of the column of the fornix [1, 2, 5]. For this reason, the rare occurrence of aneurysms with anatomical variants of the ACA–ACoA complex is crucial to knowing and avoiding surgical complications.

On the other hand, aneurysms threatening the posterior cerebral circulation represent defiant entities situated in narrow corridors near vital structures. For instance, basilar aneurysms represent about 5%–8% of all intracranial aneurysms and are associated with high morbidity and mortality [13, 14]. Over the years, they have represented significant challenges for endovascular and microsurgical approaches, bringing great controversy about their management [15, 16].

In this case study, we report the uncommon presentation of a patient with a distal fusiform aneurysm arising from an accessory A2 artery coexisting with a basilar aneurysm. As underscored in the operative video, we describe surgical nuances that accounted for the optimized microsurgical management of both entities via a single craniotomy.

## Case presentation

A 58-year-old male was referred to our emergency department with a 3-day history of sudden onset headache, loss of consciousness, and uncontrolled vomiting. His personal medical history was relevant for chronic hypertension. Upon neurological examination, the patient had 3/5 quadriparesis and a Glasgow coma scale (GCS) score of 8, which merited neurocritical intensive care.

**Figure 1:**
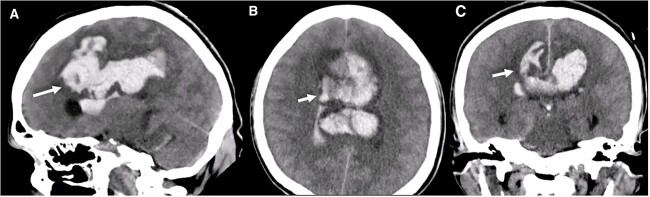
Brain CT without contrast shows a large interhemispheric hematoma, intraventricular hemorrhage, and subarachnoid hemorrhage (white arrows) in (A) Sagittal, (B) Axial, and (C) Coronal views.

**Figure 2:**
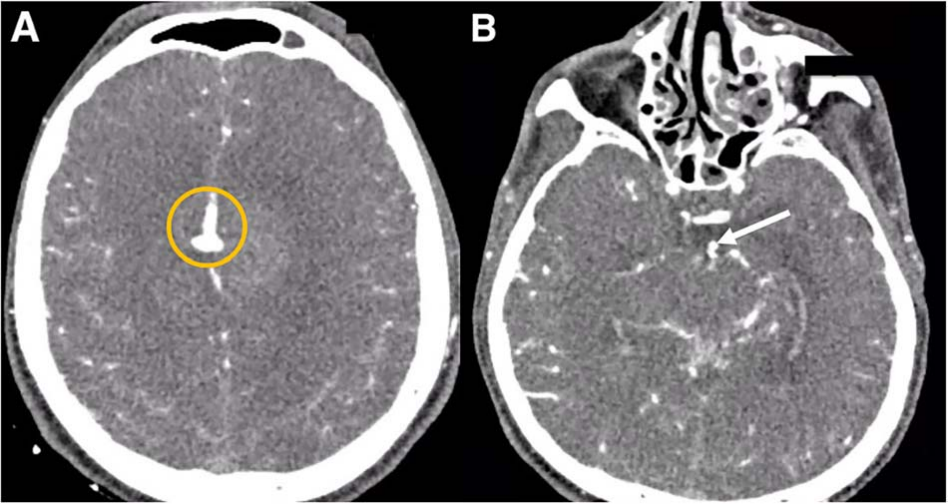
Cerebral angiotomography. (A) Axial cut depicts a midline irregular vascular lesion (yellow circle). (B) Axial cut shows a small saccular aneurysm at the basilar artery (white arrow).

**Figure 3:**
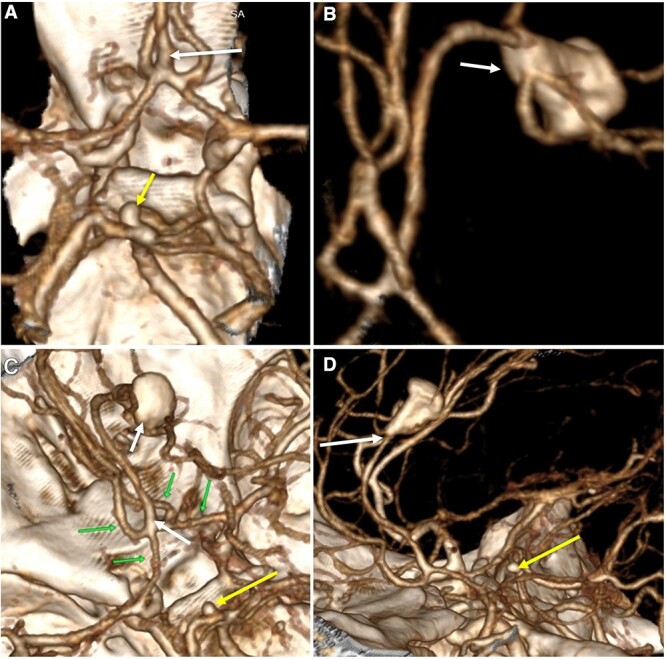
Three-dimensional reconstruction imaging. (A) Superior view of the Circle of Willis shows a variant of the ACA with an accessory A2 branch (white arrow) and a saccular aneurysm of 3.3 × 2.8 mm at the basilar artery (yellow arrow). (B) Inferior–superior view depicts the configuration of the ACA with a triplicated A2 segment and a fusiform aneurysm of 12 × 7 mm emerging in its distal portion, with branches arising from the aneurysm sac (white arrow). (C) Superior view of the anterior and posterior circulation exhibits the complex anatomy of the A1 and A2 segments (green arrows), the accessory A2 segment and distal fusiform aneurysm (white arrows), and the basilar aneurysm (yellow arrow). (D) Lateral view shows the course of the triplicated A2 segment with the fusiform aneurysm arising distally (white arrow) and the small basilar aneurysm (yellow arrow).

A multislice computed tomography (CT) without contrast revealed an extensive interhemispheric hematoma in the frontoparietal region, subarachnoid hemorrhage, and intraventricular hemorrhage in the adjacent area (Fisher grading score of IV) (Fig. 1). In addition, cerebral angiotomography and 3D reconstruction imaging suggested the presence of two abnormal vascular lesions: (i) a fusiform aneurysm of 12 × 7 mm in an accessory A2 portion of the ACA and (ii) an unruptured saccular aneurysm of 3.3 × 2.8 mm in the distal segment of the basilar artery adjacent to the anterolateral surface of the P1 segment and the left superior cerebellar artery (Figs 2 and 3).

After careful appraisal of the patient’s presentation, complex aneurysms’ morphology, and initial medical management in the neurocritical care unit for 14 days, he was deemed a candidate for microsurgical management.

### Operative note

The patient was placed supine with a 30-degree head rotation to the left. Next, we performed a right pterional craniotomy with the extension of the opening toward the base of the temporal bone. After carefully dissecting the dura mater, the Sylvian fissure was exposed to access the subarachnoid space, which, in turn, permitted the visualization of the ICA and optic and oculomotor nerves. This dissection maneuver allowed the temporal pole’s mobilization without retracting the neurovascular structures and exposed the interpeduncular fossa (Video 1).

Fine dissection continued until the identification of the A1–M1 bifurcation of the ICA. Then, we visualized the right A1 segment and gained access to the carotid and chiasmatic cisterns. The brain remained relaxed, which allowed for further evaluation of the complex anatomical morphology of the ACA and ACoA. We observed an accessory branch from the ACoA and correlated its relationship visualized in the head CT. Because we had corroborated that the territory irrigated by the accessory A2 artery had collateral supply from both ACA, we safely clipped it proximally, thus excluding the aneurysm from the anterior circulation. Concluding this first stage of surgery, fluorescein angiography verified the permeability of the anterior communicating complex (Video 1).

Subsequently, we assessed the surgical corridors and subarachnoid cistern to clip the basilar aneurysm. Next, following the course of the posterior communicating artery, we localized the basilar artery, the P1 segment of the posterior cerebral artery, and both superior cerebellar arteries. Then, we proceeded to identify and clip the aneurysm neck. Finally, fluorescein angiography confirmed the permeability of the surrounding vascular structures with the exclusion of the aneurysm (Video 1).

### Postoperative course

**Figure 4:**
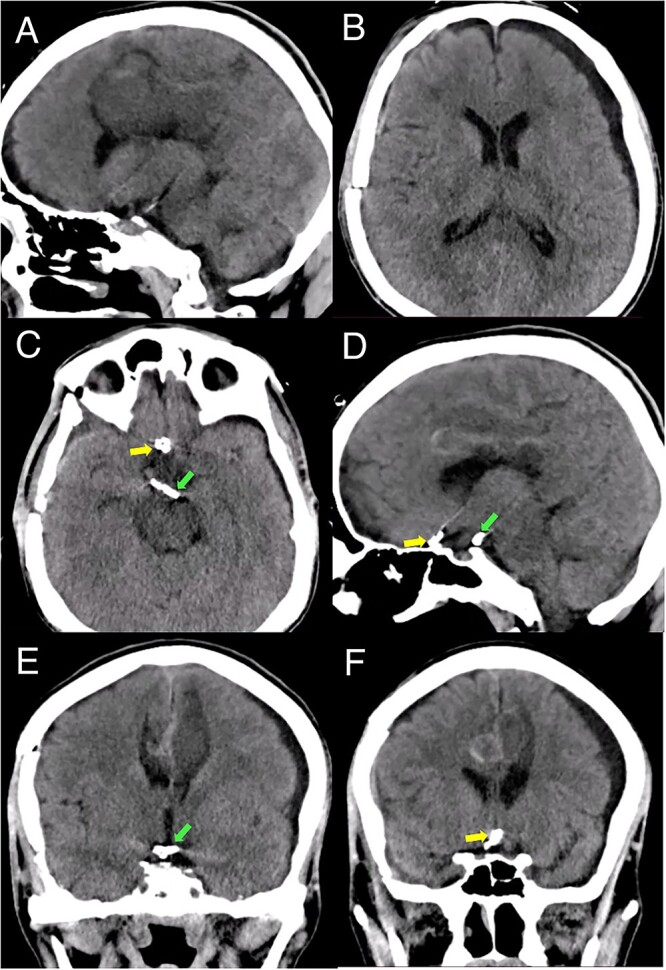
Postoperative head CT. (A, B) Sagittal and axial views show resolution of the hematoma and intraventricular hemorrhage with remaining postinfarction areas. (C–F) Postoperative head CT views depict the placement of clips in the accessory A2 artery (yellow arrow) and basilar aneurysm (green arrow).

Brain imaging demonstrated improvement of the hematoma and intraventricular hemorrhage with remnant postinfarction areas (Fig. 4). The patient was transferred to neurointensive care postoperatively, where he remained for 7 days. At this point, the neurological examination indicated he had 4/5 quadriparesis, a right third nerve paresis, and a GCS score of 13. Seven days later, he was discharged with a modified Rankin scale (mRS) score of 4. At the 1-month follow-up, the patient had a GCS of 14 and a mRS of 4. At the 3-month follow-up, the patient showed a significant improvement in the GCS score with a mRS of 2 and improved function of the right third cranial nerve.

## Discussion

Brain aneurysms are a highly morbid and mortal condition [14]. Treatment strategies have evolved over the years to provide optimal outcomes. However, aside from the patient’s clinical condition, aneurysm’s morphology, and clinical settings, there are particular challenges with approaching these entities, one of which represents the associated anatomical variations of brain vessels [3, 6, 8]. Critically important is the recognition of anatomical variations in the ACA–ACoA complex to avoid ischemic and detrimental clinical outcomes.

The presence of a triplicate A2 artery is not uncommon; however, the presence of aneurysms around this vessel variant is rare [6–12]. Few cases describing aneurysms in the accessory A2 artery have been published, most of them treated surgically and yielding good neurological outcomes [5, 11, 12, 17–30]. Jalali *et al*. studied 36 patients undergoing aneurysm treatment, of which seven (19.4%) posed the triple-A2 variant. Interestingly, patients with ACoA aneurysms had a significantly higher prevalence of the triple-A2 variant compared with the general population (*P* < 0.00001) [2]. Moreover, the authors described that patients with ACoA aneurysms that bore the triple-A2 variant were more likely to be treated via open surgery (71%) versus endovascular procedures (29%) [2]. In another study analyzing seven distal accessory ACA aneurysms, Sirin *et al*. suggested that clipping was the favored treatment modality, and the outcomes were excellent in 86% of cases [5]. On the other hand, it is worth noting that even rarer has been the report of a fusiform subtype of accessory ACA aneurysms, like our illustrated case.

In the presented study, a comprehensive understanding of the anatomic variations around the ACoA–ACA complex could not have been underestimated for achieving a safe approach. As depicted in the surgical video, diligently appraising the anatomical configuration was paramount to properly secure the clipping of the distal fusiform aneurysm in the accessory A2 segment of the ACA. Moreover, the assessment of the surgical corridors and the team’s technical expertise accounted for clipping the basilar aneurysm in a single craniotomy.

## Conclusion

Aneurysms of the accessory A2 artery are rare entities that demand nuanced acquaintance with anatomical variations in the anterior circulation. In addition, basilar aneurysms are situated in a narrow corridor near vital perforators and complex anatomy. Both vascular pathologies require dexterity and expertise to provide a safe treatment approach.

The presented case illustrated the uncommon and challenging occurrence of an accessory A2 artery aneurysm coexisting with a basilar aneurysm. Notably, recognizing the anatomy of a vascular variant, its blood supply, and the surgical team’s expertise with pathologies of narrow corridors were of utmost importance in providing safe treatment.

## Conflict of interest statement

None declared.

## Funding

None declared.

## Supplementary Material

A2_and_Basilar_aneurysms_rjad742

## References

[1] Baptista AG . Studies on the arteries of the brain: II. The anterior cerebral artery: some anatomic features and their clinical implications. Neurology 1963;13:825. 10.1212/WNL.13.10.825. 14066997

[2] Jalali A , Srinivasan VM, Kan P, Duckworth EAM. Association of Anterior Communicating Artery Aneurysms with triplicate A2 segment of the anterior cerebral artery. World Neurosurg 2020;140:e234–9. 10.1016/j.wneu.2020.05.005. 32407912

[3] Dunker RO , Harris AB. Surgical anatomy of the proximal anterior cerebral artery. J Neurosurg 1976;44:359–67. 10.3171/jns.1976.44.3.0359. 1249614

[4] Parmar H , Sitoh YY, Hui F. Normal variants of the intracranial circulation demonstrated by MR angiography at 3T. Eur J Radiol 2005;56:220–8. 10.1016/j.ejrad.2005.05.005. 15950421

[5] Sirin A , Cikla U, Uluc K, Baskaya MK. Ruptured distal accessory anterior cerebral artery aneurysm: a case report and literature review. Turk Neurosurg 2018;28:839–42. 10.5137/1019-5149.JTN.20424-17.1. 28944940

[6] Krabbe-Hartkamp MJ , van der Grond J, de Leeuw FE, de Groot JC, Algra A, Hillen B, et al. Circle of Willis: morphologic variation on three-dimensional time-of-flight MR angiograms. Radiology 1998;207:103–11. 10.1148/radiology.207.1.9530305. 9530305

[7] Krzyżewski RM , Tomaszewski KA, Kochana M, Kopeć M, Klimek-Piotrowska W, Walocha J. Anatomical variations of the anterior communicating artery complex: gender relationship. Surg Radiol Anat 2014;37:81–6. 10.1007/s00276-014-1313-7. 24849465 PMC4295032

[8] Uchino A . Variations of the proximal anterior cerebral artery (ACA), including anterior communicating artery (ACoA). Atlas Supraaortic Craniocervical Arter Var 2022;109–30. 10.1007/978-981-16-6803-6_9.

[9] Ješić A , Torbica S, Marić S, Popović S, et al. Anatomic variations of the anterior portion of the circle of Willis: an MR angiography study. Curr Top Neurol Psychiatr Relat Discip 2011;XIX:9–16.

[10] Gomes FB , Dujovny M, Umansky F, Berman SK, Diaz FG, Ausman JI, et al. Microanatomy of the anterior cerebral artery. Surg Neurol 1986;26:129–41. 10.1016/0090-3019(86)90365-4. 3726739

[11] Nathal E , Yasui N, Sampei T, Suzuki A. Intraoperative anatomical studies in patients with aneurysms of the anterior communicating artery complex. J Neurosurg 1992;76:629–34. 10.3171/jns.1992.76.4.0629. 1545257

[12] Ogawa A , Suzuki M, Sakurai Y, Yoshimoto T. Vascular anomalies associated with aneurysms of the anterior communicating artery: microsurgical observations. J Neurosurg 1990;72:706–9. 10.3171/jns.1990.72.5.0706. 2324796

[13] Tjahjadi M , Serrone J, Hernesniemi J. Should we still consider clips for basilar apex aneurysms? A critical appraisal of the literature. Surg Neurol Int 2018;9:44. 10.4103/sni.sni_311_17. 29541485 PMC5843972

[14] Brisman JL , Song JK, Newell DW. Cerebral aneurysms. N Engl J Med 2006;355:928–39. 10.1056/NEJMra052760. 16943405

[15] Adeeb N , Ogilvy CS, Griessenauer CJ, Thomas AJ. Expanding the indications for flow diversion: treatment of posterior circulation aneurysms. Neurosurgery 2019;86:S76–84. 10.1093/neuros/nyz344. 31838535

[16] Medani K , Hussain A, Quispe Espíritu JC, Mayeku J, Avilés-Rodríguez GJ, Sikka A, et al. Basilar apex aneurysm systematic review: microsurgical versus endovascular treatment. Neurochirurgie 2022;68:661–73. 10.1016/j.neuchi.2022.07.007. 35965246

[17] Jie H , Jin-Qing H, Cheng-Chuan J, Xiao-Qiang W. Ruptured aneurysm at the origin of the median artery of the corpus callosum: case report. J Neurol Sci 2012;28:632–6.

[18] Aso K , Kashimura H, Matsumoto Y, Saura H. Microsurgical clipping for anterior communicating artery aneurysm associated with the accessory anterior cerebral artery via the pterional approach. Surg Neurol Int 2018;9:120. 10.4103/sni.sni_103_18. 30009084 PMC6024506

[19] Matsuzaki K , Uno M, Fujihara T, et al. Ruptured distal accessory anterior cerebral artery aneurysm. Neurol Med Chir 2011;51:839–42. 10.2176/nmc.51.839. 22198106

[20] Morigaki R , Uno M, Matsubara S, Satoh K, Nagahiro S. Choreoathetosis due to rupture of a distal accessory anterior cerebral artery aneurysm. Cerebrovasc Dis 2008;25:285–7. 10.1159/000119640. 18332626

[21] Javier DG , Ricardo MM, Alan HH, et al. Median artery of the corpus callosum in the context of anterior communicating artery aneurysm rupture: the relevance of perianeurysmal anatomy. Arch Neurocienc 2023;28:44–8. 10.31157/an.v28i2.421.

[22] Ładziński P , Maliszewski M, Majchrzak H. The accessory anterior cerebral artery: case report and anatomic analysis of vascular anomaly. Surg Neurol 1997;48:171–4. 10.1016/S0090-3019(96)00421-1. 9242244

[23] Kwak R , Niizuma H, Hatanaka M, Suzuki J. Anterior communicating artery aneurysms with associated anomalies. J Neurosurg 1980;52:162–4. 10.3171/jns.1980.52.2.0162. 7351555

[24] Seo DH , Lee WC, Choe IS, Park SC, Ha YS. Ruptured and unruptured aneurysms of the accessory anterior cerebral artery combined with a blood blister-like aneurysm of the anterior communicating artery. Neurol India 2009;57:85–7. 10.4103/0028-3886.48806. 19305088

[25] Agrawal A , Jagetia A, Bodeliwala S, Singh D, Dutta G, Shah A. Intraoperative microsurgical anatomy of the anterior communicating artery complex harbouring an anterior cerebral territory aneurysm. Neurol India 2019;67:823–8. 10.4103/0028-3886.263174. 31347561

[26] Morioka M , Fujioka S, Itoyama Y, Ushio Y. Ruptured distal accessory anterior cerebral artery aneurysm: case report. Neurosurgery 1997;40:399–402. 10.1097/00006123-199702000-00036. 9007878

[27] Aydin IH , Takçi E, Kadioğlu HH, Tüzün Y, Kayaoğlu CR, Barlas E. Vascular variations associated with anterior communicating artery aneurysms-an intraoperative study. Minim Invasive Neurosurg 1997;40:17–21. 10.1055/s-2008-1053407. 9138303

[28] Moon M , Jang DK, Cho BR. Alternate simultaneous bilateral carotid angiography in Y-stent−assisted coil embolization for an anterior communicating artery aneurysm with triplicate A2 variant. World Neurosurg 2023;170:38–42. 10.1016/j.wneu.2022.11.106. 36464155

[29] Takahashi S , Ohnaka K, Ishiwada T, Kino T, Arai T. Infarction of the entire corpus callosum as a complication in subarachnoid hemorrhage: a case report. Interdiscip Neurosurg 2017;7:53–5. 10.1016/j.inat.2016.12.005.

[30] Martínez F , Spagnuolo E, Calvo-Rubal A, Laza S, Sgarbi N, Soria-Vargas VR, et al. Variaciones del sector anterior del polígono de Willis. Correlación anatomo-angiográfica y su implicancia en la cirugía de aneurismas intracraneanos (Arterias: ácigos cerebral anterior, mediana del cuerpo calloso y cerebral media accesoria). Neurocirugia 2004;15:578–88. 10.1016/S1130-1473(04)70449-2. 15632994

